# The epidemiology of medical emergency contacts outside hospitals in Norway - a prospective population based study

**DOI:** 10.1186/1757-7241-18-9

**Published:** 2010-02-18

**Authors:** Erik Zakariassen, Robert Anders Burman, Steinar Hunskaar

**Affiliations:** 1 National Centre for Emergency Primary Health Care, Uni Health, Bergen, Norway, Kalfarveien 31, 5018 Bergen, Norway; 2 Department of Research, Norwegian Air Ambulance Foundation, Post Box 94, 1441, Drøbak, Norway; 3 Section for General Practice, Department of Public Health and Primary Health Care, University of Bergen, Post Box 7804, 5020 Bergen, Norway

## Abstract

**Introduction:**

There is a lack of epidemiological knowledge on medical emergencies outside hospitals in Norway. The aim of the present study was to obtain representative data on the epidemiology of medical emergencies classified as "red responses" in Norway.

**Method:**

Three emergency medical dispatch centres (EMCCs) were chosen as catchment areas, covering 816 000 inhabitants. During a three month period in 2007 the EMCCs gathered information on every situation that was triaged as a red response, according to The Norwegian Index of Medical Emergencies (Index). Records from ground ambulances, air ambulances, and the primary care doctors were subsequently collected. International Classification of Primary Care - 2 symptom codes (ICPC-2) and The National Committee on Aeronautics (NACA) Score System were given retrospectively.

**Results:**

Total incidence of red response situations was 5 105 during the three month period. 394 patients were involved in 138 accidents, and 181 situations were without patients, resulting in a total of 5 180 patients. The patients' age ranged from 0 to 107 years, with a median age of 57, and 55% were male. 90% of the red responses were medical problems with a large variation of symptoms, the remainder being accidents. 70% of the patients were in a non-life-threatening situation. Within the accident group, males accounted for 61%, and 35% were aged between 10 and 29 years, with a median age of 37 years. Few of the 39 chapters in the Index were used, A10 "Chest pain" was the most common one (22% of all situations). ICPC-2 symptom codes showed that cardiovascular, syncope/coma, respiratory and neurological problems were most common. 50% of all patients in a sever situation (NACA score 4-7) were > 70 years of age.

**Conclusions:**

The results show that emergency medicine based on 816 000 Norwegians mainly consists of medical problems, where the majority of the patients have a non-life-threatening situation. More focus on the emergency system outside hospitals, including triage and dispatch, and how to best deal with "everyday" emergency problems is needed to secure knowledge based decisions for the future organization of the emergency system.

## Introduction

Persons in need of acute medical assistance are supposed to come in contact with the emergency care system by calling a three digits emergency number (113) to an emergency medical dispatch centre (EMCC). The 19 EMCCs are responsible for alarming the out-of-hospitals emergency resources like ambulances services (ground and air) and primary care doctors on-call.

For all calls to an EMCC, trained nurses use The Norwegian Index of Medical Emergencies (Index) [1] to classify the medical problem into one of three different levels of response; green, yellow and red, the latter indicating immediate need of help (potentially or a manifest life-threatening situation). When an emergency situation is classified as red, there will be transmitted a simultaneous radio alarm from the EMCC to doctors on-call and the ambulances in the relevant area.

Even though emergency medicine is considered an important part of the health care system, little is known about the incidence and management of medical emergencies outside hospitals in Norway. Emergency medicine is not a formal speciality for doctors in Norway. Still, treatment of critically ill or injured people is defined as emergency medicine. Earlier white papers and plans concerning the organisation of the emergency services underscore the lack of national statistics and scarce epidemiological knowledge [2-4]. It has for long been anticipated a rate of about 10 red responses per 1 000 inhabitants per year, but this figure has not been supported by valid statistics or scientific studies [3]. Data from a representative sample of Norwegian out-of-hours districts showed a rate of 9 red responses per 1 000 inhabitants per year, but this number was based on data from local emergency communication centres, not EMCCs [5,6]. A recent study from a single island municipality with approximately 4 000 inhabitants found an incidence of 27 medical emergencies per 1 000 inhabitants per year [7]. However, the definition of an emergency was wider in this study than the classification of a red response based on the Index of Medical emergencies from EMCCs.

There seems to be a scarce literature with broad epidemiological approach to pre-hospital emergencies in general. Most studies deal with specific emergency problems like cardiac arrest, chest pain or trauma [8-14]. One study in Norway has a wider epidemiological scope [7]. More epidemiological knowledge is needed to make the right decisions for policy makers and leaders of the health care services.

To obtain representative data on the epidemiology of medical emergencies classified as "red response" by the EMCCs, we performed a large prospective population based study.

## Materials and methods

For data collection we chose and cooperated with a strategic sample of three EMCCs, located at Haugesund, Stavanger and Innlandet hospitals, covering Rogaland, southern part of Hordaland, Hedmark, and Oppland counties, covering a total of 69 581 km^2 ^(21% of the total area ofNorway) and 816 000 inhabitants (18% of the total population). Data registration was performed prospectively during a period of three months, from October 1^st ^to December 31^st ^2007.

### Variables

All EMCCs use a software system called Acute Medical Information System (AMIS) to record all incoming situations. Usage of the AMIS system results in an electronic form with registration of each incident (not the individual patient). The AMIS form contains basic information about the situation, the patient(s), all available logistics (date, time registration for incoming alarm and all alarms and electronic messages sent to the different prehospital resources, who responded and when), and to where the patients are transported (left at scene, home, casualty clinic, hospital).

Based on the immediate available information, the EMCC operator (usually a specially trained nurse) gives the situation a clinical criteria code with a response level based on the Index [1]. The Index is based on ideas from the Criteria Based Dispatch system in the US [15], and was first published in 1994. Clinical symptoms, findings and situations are categorised into 39 chapters. Each chapter is subdivided into a red, yellow and green criteria based section, correlating to the appropriate level of response. Red colour is defined as an "acute" response, with the highest priority. Yellow colour is defined as an "urgent" response, with a high, but lower priority. Green colour is defined as a "non-urgent" response, with the lowest priority.

Copies of all AMIS forms involving situations classified as red responses were sent the project manager every second week throughout the study. The EMCCs also sent copies of ambulance records from all red responses which involved ground or boat ambulances. In situations where doctors on-call or air ambulances had been involved, copies of medical records were requested by mail from the project manager directly to the person or agency involved. Several reminders were needed during collection of medical records from different parts of the health care system and continued until October 2008. To secure a uniform recording of the variables in the AMIS program, a meeting between the persons in charge of the participating EMCCs was held.

Based on information from all AMIS forms and medical records we classified the situations according to the International Classification of Primary Care - 2 (ICPC - 2) [16]. The ICPC-2 is structured into 7 components and 17 chapters from A to Z depending on the body system to which the problem belongs (table 1).

**Table 1:** International Classification of Primary Care (ICPC)

ICPC	Body system
A	General and unspecified
B	Blood, blood-forming organs, lymphatic, spleen
D	Digestive
F	Eye
H	Ear
K	Circulatory
L	Musculoskeletal
N	Neurological
P	Psychological
R	Respiratory
S	Skin
T	Endocrine, metabolic and nutritional
U	Urology
W	Pregnancy, childbearing, family planning
X	Female genital system
Y	Male genital system
Z	Social problems

Component 1 (codes -01 to -29) provides codes for symptoms and complaints. The analyses in this study were based on codes from the symptom component solely. Each patient was given one code only (e.g. D01 for abdominal pain or N07 for convulsions). For further analyses the symptom-codes were aggregated into clinically connected and appropriate groups based on the chapters from A to Z. ICPC codes were classified in medical records from the doctors on-call. All other ICPC codes were classified by two members of the research team with experience in emergency medicine. Main symptom was used for ICPC coding

Based on all available information according to The National Committee on Aeronautics (NACA) Score System [17], the severity of the medical problem was classified (table 2).

**Table 2:** National Committee on Aeronautics (NACA)

Score level	Patient status
NACA 0	No injury or illness
NACA 1	Not acute life-threatening disease or injury
NACA 2	Acute intervention not necessary; further diagnostic studies needed
NACA 3	Severe but not life threatening disease or injury; acute intervention necessary
NACA 4	Development of vital (life threatening) danger possible
NACA 5	Acute vital (life threatening) danger
NACA 6	Acute cardiac or respiratory arrest
NACA 7	Death

The NACA score system was chosen because it is easy to use retrospectively and the air ambulances use NACA score as a routine for their patients. The patient's status is classified from 0 to 7, zero indicating no disease or injury, while seven indicates the patient being dead. NACA score was in the analyses categorised as NACA 0-1, indicating a patient either with no symptoms/injuries or in no need of medical treatment, NACA 2-3, indicating need of medical help where value 3 indicates need of hospitalisation, but still not a life-threatening situation. NACA 4-6 indicates potentially (4) and definitely life-threatening medical situations (5 and 6) and NACA 7 is a dead person. NACA scores were classified prospectively in patients transported by air ambulance, and the scores were found in the medical records. All other NACA scores were classified by two members of the research team with experience in emergency medicine. In case of multi-patient accidents the most severely injured patient was included from each situation.

### Statistical analyses

The statistical analyses were performed using Statistical Package for the Social Sciences (SPSS version 15). Standard univariate statistics were used to characterise the sample. Skewed distributed data are presented as median with 25-75% percentiles. Rate is presented as numbers of red responses per 1 000 inhabitants per year with a 95% confidence interval (CI). A p-value of < 0.05 was considered significant. Index categories were merged into the five most used (A01/A02 "Unconscious", A05 "Ordered mission", A06 "Inconclusive problem", A10 "Chest pain" and A34/A35 "Accidents") and one category containing the rest, called "All Other" in the analyses. In the analysis of diurnal variations, NACA scores were dichotomised to non life-threatening or life-threatening situations. In 64 patients we were not able to extract information on gender, patients' whereabouts in 82 situations and where patients where brought to in 50 situations. In 435 situations it was not possible to decide NACA score and in 39 situations ICPC symptoms score.

### Ethics and approvals

Approval of the study was given by the Privacy Ombudsman for Research, Regional Committee for Medical Research Ethics, and the Norwegian Directorate of Health.

## Results

**Figure 1:**
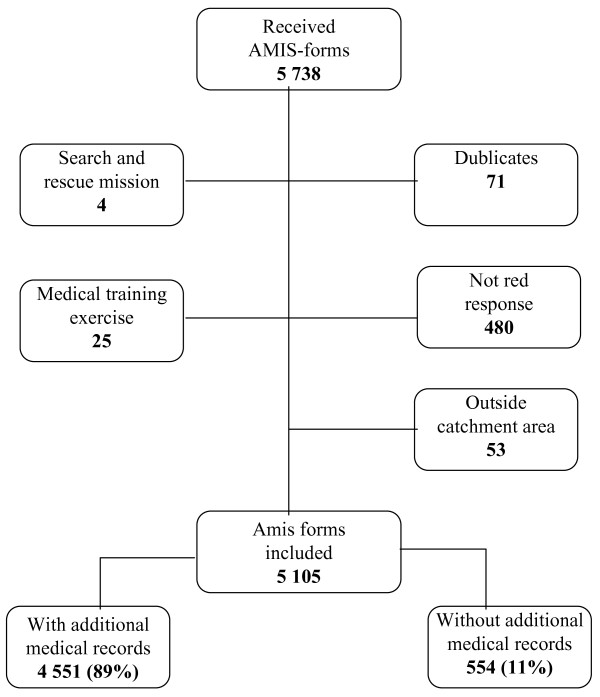
**Is a flow chart of total collected, excluded and included AMIS forms**.

The three participating EMCC-districts collected 5 738 AMIS forms for the study, of which 633 were excluded, due to e.g. situations not being red responses and duplicates (fig 1).

Total incidence of red response situations was then 5 105 during the three month period corresponding to a rate of 25.1 (24.4-25.7) situations per 1 000 inhabitants per year. Innlandet had a rate of 30.6 (29.4-31.8), Stavanger 20.0 (19.0-21.0) and Haugesund 22.9 (21.4-24.3) Differences in rates between the three EMCC areas was all statistically significant (p < 0.000). In 104 situations the mission was aborted (no patients), six situations concerned allocation of ambulance resources (no patients) and 71 situations were support to other emergency units (fire and police departments, no patients). 394 patients were involved in 138 accidents, resulting in 256 more patients than situations in which 77 situations had 2 patients, 30 situations had 3 patients, and 16, 9 and 6 situations had 4, 5 and 6 or more patients, respectively. The total number of patients was 5 180 which corresponds to a rate of 25.5 (24.7-26.1) patients per 1 000 inhabitants per year. Of the 256 extra patients from the accidents, 98% had a NACA score of 3 or lower, one was dead. The 256 extra patients, all interrupted missions, allocations of ambulances, and support to other emergency units were excluded from further statistical analyses, and the material thus consists of the remaining 4 924 red response situations with the same number of patients.

### Demography and Index categories

The patients' age ranged from 0 to 107 years, with a median age of 57 (33-75). The gender distribution showed 55% men with median age 55, and 45% women with median age 58. Table 3 shows the five most common Index categories. The mostly used Index category was A10 "Chest pain" for both genders, and more than 80% of the patients with chest pain were over the age of 50. Index category A34/A35 "Accidents" constituted 12%, where 35% of the patients were between 10 and 29 years, and males accounted for 61%.

**Table 3:** The most frequent used Index categories by patients' gender, age, whereabouts and to where the patients were brought.

	A01/02 Unconscious		A05 Ordered mission*		A06 Inconclusive problem		A10 Chest pain		A34/35 Accidents		All other categories		Total	
	**n**	**%**	**n**	**%**	**n**	**%**	**n**	**%**	**n**	**%**	**n**	**%**	**n**	**%**

Patients	410	8	864	18	707	14	1 098	22	565	12	1 280	26	4 924	100
														
*Male*														
0-9 years	11	6	44	24	24	14	2	1	15	8	85	47	181	100
10-29 years	34	8	55	14	58	14	13	3	119	30	123	31	402	100
30-49 years	38	7	80	15	70	13	111	21	97	19	128	25	524	100
50-69 years	62	7	133	16	132	16	275	33	70	9	158	19	830	100
> 70 years	81	11	126	18	131	18	211	29	32	5	139	19	720	100
Total	226	9	438	16	415	16	612	23	333	12	633	24	2 657	100
														
*Female*														
0-9 years	20	16	20	16	11	10	1	1	8	6	63	51	123	100
10-29 years	28	8	56	16	39	11	12	3	76	21	151	42	362	100
30-49 years	29	7	80	19	55	13	67	16	50	12	152	35	433	100
50-69 years	23	5	81	17	75	15	156	32	45	9	110	23	490	100
> 70 years	77	10	171	21	110	14	249	31	31	4	157	20	795	100
Total	177	8	408	19	290	13	485	22	210	9	633	29	2 203	100
														
*Patients' whereabouts*														
At home	243	9	349	12	416	15	833	30	87	3	882	31	2 810	100
Casualty clinic	4	3	115	77	3	2	17	11	1	1	10	6	150	100
Doctor's surgery	2	1	105	54	4	2	62	32	4	2	19	9	199	100
Public area	113	9	65	6	221	19	94	8	442	37	249	21	1 184	100
Hospitals	0	0	137	87	0	0	9	6	0	0	11	7	157	100
Nursing home	22	9	64	27	34	15	51	22	2	1	60	26	233	100
Other	13	12	12	11	21	19	20	18	15	14	29	26	110	100
Total	397	8	849	18	699	15	1 086	22	551	11	1 260	26	4 842	100
														
*Patients brought to*														
Casualty clinic	57	8	76	10	151	21	155	21	105	14	187	26	731	100
Hospital via casualty clinic	27	5	76	15	100	19	127	24	52	10	138	27	520	100
Directly hospital, doctor involved	107	6	544	32	145	8	424	25	159	9	337	20	1 716	100
Directly hospital, doctor not involved	102	9	87	7	175	15	274	23	175	15	364	31	1 177	100
Remained on site	42	8	55	11	82	16	100	19	43	8	200	38	522	100
Deceased	64	38	12	7	37	22	10	6	14	9	30	18	167	100
Taken care of by other	5	12	3	7	11	27	2	5	8	20	12	29	41	100
Total	404	8	853	18	701	15	1 092	22	556	11	1 268	26	4 874	100

The variables have some missing data and the total may not add up to 4 924 for all groups.

* Mission ordered by health personnel or other emergency units, i.e. transport directly to hospital or ambulance assistance to other emergency

The incidence of red responses was higher during daytime (0800-1529) compared to night time (2300-0759) for most of the Index categories, except for category "all other" which had only minor skewness around the clock (table 4). A34/A35 "Accidents" showed the highest incidence during daytime with a proportion of 45% (table 4).

**Table 4:** The most frequent used Index categories by time of day and NACA-score.

	A01/02 Unconscious		A05 Ordered mission		A06 Inconclusive problem		A10 Chest pain		A34/35 Accidents		All other categories		Total	
	**n**	**%**	**n**	**%**	**n**	**%**	**n**	**%**	**n**	**%**	**n**	**%**	**n**	**%**

*Time of day*														
														
0800-1529	170	41	367	43	275	39	393	36	256	45	439	34	1 897	39
1530-2259	137	34	292	34	266	38	368	34	211	38	447	35	1 721	35
2300-0759	103	25	199	23	160	23	332	30	97	17	388	31	1 279	26
Total	410	100	858	100	701	100	1 093	100	561	100	1 274	100	4 897	100
														
*NACA-score*														
														
0-1	38	10	44	6	95	15	87	9	101	19	86	7	451	10
2-3	163	43	465	59	418	65	631	65	326	62	747	63	2 750	61
4-6	117	30	265	34	96	15	243	25	83	16	318	27	1 122	25
7	64	17	11	1	37	5	10	1	14	3	30	3	166	4
Total	382	100	785	100	646	100	971	100	524	100	1 118	100	4 489	100

Due to some missing data total numbers will not add up to 4 924 for all groups.

A29 "Breathing difficulties" was the most used Index-category in the "all other" group with nearly 5% of the total. Approximately half of all patients in the youngest age group had "all other" medical problems and convulsions (A23) was the most common Index category with 14% of the situations. Seven Index categories were each used five times or less and six were not used at all.

### Severity of injury and illness

NACA-score could be set in 4 489 (91%) of the 4 924 situations with patients (table 4). Males constituted 68% of the 246 patients with NACA 6-7. Patients >70 years accounted for 50% of the 1 280 patients with potentially/manifest life-threatening medical situations pronounced dead (NACA 4 and higher). Median age of the dead patients was 69 (53-81).

More than 60% of the patients were in category NACA 2-3. Also a large majority of the accidents (81%) were given NACA-score 0-3, indicating non-life threatening situations. Considering the 166 patients that were pronounced dead on arrival or resuscitated without return of spontaneous circulation (NACA 7), 64 (39%) were given the code A01/A02 "Unconscious", 37 (22%) A06 "Inconclusive problem", 14 (8%) A34/A35 "Accidents", and 10 (6%) A10 "Chest pain". The percentage of patients with non life-threatening conditions increased from 70% at daytime to 74% at night, while life-threatening conditions decreased from 30% at daytime to 26% at night. Differences in NACA distribution between the districts were not statistical significant (p > 0.05).

### Patients' whereabouts and final level of care

Table 3 also describes the patients' whereabouts and where the patients were brought, by Index categories. Overall, 58% of the 4 924 patients were residing at home or at private facilities, while one fourth were in public areas. The primary health care services (casualty clinics, doctors' surgeries and nursing homes) constituted 12% of the patients' whereabouts. 77% of the situations with A10 "Chest pain" were in private homes and 80% of the situations with A34/A35 "Accidents" were in public places.

A total of 3 413 (70%) patients were brought to a hospital, either via the casualty clinic (11%) or directly with (35%) or without (24%) being examined by a doctor first. Patients who remained on site accounted for 11% of the patients. The table also shows that in 26% of the situations, the casualty clinics were directly involved in patient care, either as final place of treatment or by examination and subsequent referrals to hospital. Considering the accidents alone, 28% of the 556 patients were brought to a casualty clinic. Among the 77 patients with diabetes as the main cause of contact with the EMCC, 73% remained on site after treatment.

### ICPC symptom score

In 4 551 (92%) patients we retrieved one or more medical record, and in 99% of all patients a symptom-code was registered. Table 5 shows the symptom distribution where 89% had medical symptoms, while injuries/traumas accounted for 11% of the patients. Cardiovascular symptoms was the most common symptom group (N = 1 389, 28%), and loss of consciousness second, accounting for 945 of the situations (19%). Chest pain or pain related to the heart dominated the cardiovascular patients with 95%. Of the 465 patients categorised under "Other", 23% had a problem related to pregnancy or labour.

**Table 5:** Patient distribution according to the ICPC-2 classification system with frequencies, rate and national estimate per year

ICPC symptoms	ICPC-code (n)	N	%	Rate per 1000/year	National estimate/year
Cardiovascular		1 389	28	6.8	31 100
Chest/heart pain	A11 (808) K01 (513)				
Other cardiovascular symptoms	K29 (68)				
					
Loss of consciousness		945	19	4.6	21 200
Syncope/coma	A06/07 (945)				
					
Respiratory		472	10	2.3	10 600
Dyspnoea/breathing problems	R02/04 (430)				
Other respiratory symptoms	R29 (42)				
					
Neurological		592	11	2.9	13 300
Convulsion	N07 (324)				
Other neurological symptoms	N29 (268)				
					
Digestive		195	4	1.0	4 400
Abdominal pain/cramps	D01 (113)				
Other digestive symptoms	D29 (82)				
					
Psychiatric		296	6	1.5	6 600
Acute alcohol abuse	P16 (113)				
Other psychiatric symptoms	P29 (182)				
					
Injury/trauma		531	11	2.6	11 900
Laceration/cut, skin	S18 (101)				
Other skin symptoms other	S29 (34)				
Other musculoskeletal symptoms	L29 (396)				
					
Other		465	10	2.3	10 400
Endocrine/metabolic symptoms	T29 (11)				
Urinary/male genital symptoms	U29 (7) Y29 (5)				
Pregnancy/female genital symptoms	W29 (106) X29 (1)				
Assault/harmful event/problem	Z25 (12)				
General symptoms	A29 (317)				
Eye symptoms	F29 (6)				
					
Not classified		39	1	0.2	
Subtotal		4 924	100	24.2	110 000
					
Excluded patients		256		1.3	
					
Total		5 180		25.5	116 000

Most of the symptom groups were more or less equally gender distributed for all ages, except for traumas/injuries with a large male majority (63% of the 521 situations). Cardiovascular symptoms were common among the men over the age of 30, with a peak incidence in the age group "50-69 years" (N= 346; 42%), while the female patients with cardiovascular symptoms tended to be older with a peak incidence in the age group "> 70 years" (N = 329; 42%). Traumas were most common in the age group 10-29 years, dominated by young males with 29% of the 399 situations in this group. In the youngest age group (0-9 years), neurological symptoms dominated in both genders, with 32% of the 180 situations among the boys, and 43% of the 123 situations among the girls.

Table S1; additional file 1 shows the Index categories A05 "Ordered mission" and A06 "Inconclusive problem" by gender, age and the patients' whereabouts. More than a third of the patients with code A05 had cardiovascular symptoms, while the symptom "Injury/trauma" (6%) was used the least. For gender there were only minor differences between the symptom groups.

## Discussion

Based on our comprehensive, prospective and population based study, estimated rate of red response patients was about 25 per 1 000 inhabitants per year in Norway. However, differences in rates between the three districts were pronounced. Index category A10 "Chest pain" was the most used category (22%), while A34/A35 "Accidents" accounted for 12% of the total. More than 70% of all red responses were found to be non life-threatening situations with NACA score = 3. Nearly 60% of the patients were at home or other private facilities. 70% of the patients were brought to hospitals, 24% of them without being examined by a doctor beforehand. One fourth of the patients were brought to a casualty clinic.

The strengths of our study include its completeness, representativity, and number of variables included. In the course of a three month period we were able to prospectively collect a complete material of more than 5 000 red responses based on a population close to 820 000 inhabitants, about 20% of the Norwegian population. In nearly 90% of all situations we retrieved records from ground and air ambulances, casualty clinics, general practitioners and doctors on-call. Together with the complete set of AMIS forms, this yields a comprehensive material for analysis of the objectives of the study. There are some limitations of the study. Severity score (NACA) on patients was assessed retrospectively based on medical records and may therefore have lower accuracy (except for situations where the air ambulances had been involved and their medical records were retrieved). The presented results are based on the EMCCs' definition of an emergency based on the Index. Undertriaged patients are thus not included.

Rate of red responses in Innlandet was higher then the rates in Stavanger and Haugesund. We see no obvious explanation for this. If the percentage of NACA 4 and above was higher in Stavanger and Haugesund compared to Innlandet, it could indicate higher accuracy and a lower level of "overtriage". This was not the fact and differences in NACA distribution between the districts were not significant. The study was not designed to investigate possible differences in triage pattern between the EMCCs.

A comparable study from Norway based on 4 400 inhabitants demonstrate mainly the same distribution between the different ICPC scores. For instance, cardiovascular problems were most common with 32%, respiratory diseases 11% and psychiatric problems constituted 5% of the situations [7]. Accidents accounted for 16% of the situations [7] which is higher percentage than in our study where accidents accounted for 11%.

Patients in the age group 50 and older represented nearly 60% of all red response situations, and persons older than 70 constituted 31%. This places emphasis on some of the upcoming challenges in emergency care, both in the primary and the secondary health care system, namely an increasingly older population and therefore more pressure on the emergency systems both inside and outside hospitals. A recently published white paper emphasised this as an important challenge for the capacity and organization of the health care system in Norway [18]. In the US, the rate of ambulance use among older patients (65 years or older) was found to be four times higher than among younger patients, all levels of responses included [19].

Medical symptoms constituted 90% of all red response situations and A10 "Chest pain" was the most used Index category for a red response. Of all 39 chapters in the Index only five were used more than 8%, in which two of those represent situations where the problem was already known (A05 "Ordered mission") or the problem could not be disclosed (A06 "Inconclusive problem"). Seven of the chapters were hardly ever used and six were not used at all. A12 "Drowning" was probably not used due to season variation. To the best of our knowledge a throughout evaluation of the Index has never been performed in Norway. The necessity of 39 chapters and the content of the chapters should be evaluated. The large majority of the red responses were given a NACA score indicating non life-threatening situations. Overtriage in dispatch centres is well known and demanding on the resources involved [20-22].

ICPC-2 coding of the symptoms resulted in a large variation of symptoms where 90% were medical problems, with cardiovascular problems as the most common one. In the category A05 "Ordered mission" cardiovascular symptoms were most common, and in A06 "Inconclusive problem" loss of consciousness was the most common symptom. The latter was probably mainly due to patients with syncope where the obvious reason for loss of consciousness was regarded as unknown.

The results show that patients involved in emergency medical situations have of a large variety of medical problems, where the majority of the patients have a non life-threatening situation. The large variation of medical symptoms stands in contrast to a narrow use of the Index as a decision tool in the EMCCs. More focus towards the emergency system outside hospitals, including triage and dispatch, and how to best deal with "everyday" emergency problems is needed in Norway. The large variety of symptoms and conditions may for instance indicate a need for more diagnostic competence at the scene of the patients. Doctors on-call in the emergency primary care services has to be more involved in emergency situations. More clinical assessment up front may lead to better medical care and to more relevant transportation routes. This challenge is addressed in a plan of action for the future emergency primary health care service in Norway [23].

## Competing interests

The authors declare that they have no competing interests.

## Authors' contributions

EZ and SH planned and established the project, including the procedures for data collection, and designed the paper. EZ and RAB performed the analyses and drafted the first manuscript. All authors took part in rewriting and approved the final manuscript.

## Supplementary Material

Additional file 1 **Table S1**: Shows the Index categories A05 Ordered mission and A06 Inconclusive problem distributed by ICPC-2 symptom categories. Click here for file
