# Supplementary material for: The epidemiology of medical emergency contacts outside hospitals in Norway - a prospective population based study
Source: Scand J Trauma Resusc Emerg Med. 2010 Feb 18;18:9. doi: 10.1186/1757-7241-18-9 (PMC2836273; doi:10.1186/1757-7241-18-9)
Supplement: Additional file 1 — Table S1: Shows the Index categories A05 Ordered mission and A06 Inconclusive problem distributed by ICPC-2 symptom categories. [file 1757-7241-18-9-S1.DOC]

|  | Cardiovascular | | Syncope/coma | | Respiratory | | Neurological | | Digestive | | Psychiatric | | Injury/trauma | | Other | | Total | |
| --- | --- | --- | --- | --- | --- | --- | --- | --- | --- | --- | --- | --- | --- | --- | --- | --- | --- | --- |
| **A05 Ordered mission** | n | % | n | % | n | % | n | % | n | % | n | % | n | % | n | % | n | % |
| *Male* | 166 | 38 | 42 | 10 | 60 | 14 | 47 | 11 | 31 | 7 | 12 | 3 | 28 | 6 | 51 | 12 | 437 | 100 |
| *Female* | 138 | 34 | 33 | 8 | 54 | 13 | 49 | 12 | 23 | 6 | 14 | 3 | 18 | 4 | 77 | 19 | 406 | 100 |
| Total | 304 | 36 | 75 | 9 | 114 | 14 | 96 | 11 | 54 | 6 | 26 | 3 | 46 | 6 | 128 | 15 | 843 | 100 |
| *Age (years)* |  |  |  |  |  |  |  |  |  |  |  |  |  |  |  |  |  |  |
| 0-9 | 1 | 1 | 2 | 3 | 25 | 37 | 15 | 22 | 6 | 9 | 0 | 0 | 5 | 7 | 14 | 21 | 68 | 100 |
| 10-29 | 8 | 7 | 8 | 7 | 13 | 11 | 18 | 16 | 8 | 7 | 11 | 10 | 15 | 13 | 34 | 30 | 115 | 100 |
| 30-49 | 51 | 31 | 13 | 8 | 18 | 11 | 15 | 9 | 6 | 4 | 11 | 7 | 9 | 6 | 40 | 25 | 163 | 100 |
| 50-69 | 110 | 52 | 19 | 9 | 20 | 9 | 19 | 9 | 17 | 8 | 6 | 3 | 7 | 3 | 14 | 7 | 212 | 100 |
| >70 | 134 | 45 | 35 | 12 | 39 | 13 | 30 | 10 | 18 | 6 | 0 | 0 | 10 | 3 | 29 | 10 | 295 | 100 |
| Total | 304 | 36 | 77 | 9 | 115 | 13 | 97 | 11 | 55 | 6 | 28 | 3 | 46 | 5 | 131 | 15 | 853 | 100 |
| *Patients whereabouts* |  |  |  |  |  |  |  |  |  |  |  |  |  |  |  |  |  |  |
| At home | 120 | 35 | 36 | 10 | 48 | 14 | 45 | 13 | 21 | 6 | 13 | 4 | 14 | 4 | 46 | 13 | 343 | 100 |
| Casualty clinic | 53 | 46 | 4 | 3 | 19 | 17 | 12 | 10 | 9 | 8 | 0 | 0 | 7 | 6 | 11 | 10 | 115 | 100 |
| Doctor’s surgery | 37 | 35 | 5 | 5 | 16 | 15 | 10 | 10 | 8 | 8 | 1 | 1 | 5 | 5 | 23 | 22 | 105 | 100 |
| Public area | 9 | 14 | 13 | 21 | 4 | 6 | 8 | 13 | 1 | 2 | 10 | 16 | 7 | 11 | 11 | 17 | 63 | 100 |
| Hospitals | 57 | 42 | 4 | 3 | 12 | 9 | 16 | 12 | 12 | 9 | 0 | 0 | 7 | 5 | 28 | 21 | 136 | 100 |
| Nursing homes | 19 | 30 | 10 | 16 | 16 | 25 | 4 | 6 | 4 | 6 | 1 | 2 | 2 | 3 | 8 | 13 | 64 | 100 |
| Other | 2 | 17 | 2 | 17 | 0 | 0 | 0 | 0 | 0 | 0 | 2 | 17 | 4 | 33 | 2 | 17 | 12 | 100 |
| Total | 297 | 35 | 74 | 9 | 115 | 14 | 95 | 11 | 55 | 7 | 27 | 3 | 46 | 5 | 129 | 15 | 838 | 100 |
|  |  |  |  |  |  |  |  |  |  |  |  |  |  |  |  |  |  |  |
| **A06 Inconclusive problem** | |  |  |  |  |  |  |  |  |  |  |  |  |  |  |  |  |  |
| *Male* | 29 | 7 | 156 | 38 | 23 | 6 | 57 | 14 | 15 | 4 | 41 | 10 | 36 | 9 | 53 | 13 | 410 | 100 |
| *Female* | 17 | 6 | 123 | 43 | 18 | 6 | 43 | 15 | 10 | 3 | 25 | 9 | 19 | 7 | 33 | 11 | 288 | 100 |
| Total | 46 | 7 | 279 | 40 | 41 | 6 | 100 | 14 | 25 | 4 | 66 | 9 | 55 | 8 | 86 | 12 | 698 | 100 |
| *Age (years)* |  |  |  |  |  |  |  |  |  |  |  |  |  |  |  |  |  |  |
| 0-9 | 0 | 0 | 9 | 25 | 4 | 11 | 9 | 25 | 1 | 3 | 0 | 0 | 4 | 11 | 9 | 25 | 36 | 100 |
| 10-29 | 3 | 3 | 32 | 33 | 4 | 4 | 19 | 19 | 4 | 4 | 18 | 18 | 8 | 8 | 10 | 10 | 98 | 100 |
| 30-49 | 7 | 6 | 25 | 20 | 5 | 4 | 16 | 13 | 7 | 6 | 22 | 18 | 20 | 16 | 23 | 18 | 125 | 100 |
| 50-69 | 18 | 9 | 79 | 39 | 11 | 5 | 33 | 16 | 8 | 4 | 22 | 11 | 13 | 6 | 20 | 10 | 204 | 100 |
| >70 | 18 | 8 | 134 | 57 | 17 | 7 | 24 | 10 | 5 | 2 | 4 | 2 | 10 | 4 | 25 | 11 | 237 | 100 |
| Total | 46 | 7 | 279 | 40 | 41 | 6 | 101 | 14 | 25 | 4 | 66 | 9 | 55 | 8 | 87 | 12 | 700 | 100 |
| *Patients whereabouts* |  |  |  |  |  |  |  |  |  |  |  |  |  |  |  |  |  |  |
| At home | 34 | 8 | 167 | 41 | 29 | 7 | 60 | 15 | 11 | 3 | 30 | 7 | 23 | 6 | 56 | 14 | 410 | 100 |
| Casualty clinic | 1 | 33 | 0 | 0 | 0 | 0 | 0 | 0 | 0 | 0 | 1 | 33 | 1 | 33 | 0 | 0 | 3 | 100 |
| Doctor’s surgery | 0 | 0 | 1 | 25 | 1 | 25 | 2 | 50 | 0 | 0 | 0 | 0 | 0 | 0 | 0 | 0 | 4 | 100 |
| Public area | 7 | 3 | 83 | 38 | 6 | 3 | 29 | 13 | 10 | 5 | 34 | 15 | 27 | 12 | 24 | 11 | 220 | 100 |
| Hospitals | 0 | 0 | 0 | 0 | 0 | 0 | 0 | 0 | 0 | 0 | 0 | 0 | 0 | 0 | 0 | 0 | 0 | 100 |
| Nursing homes | 1 | 3 | 18 | 53 | 4 | 12 | 6 | 18 | 0 | 0 | 0 | 0 | 2 | 6 | 3 | 9 | 34 | 100 |
| Other | 3 | 14 | 6 | 29 | 1 | 5 | 3 | 14 | 3 | 14 | 1 | 5 | 1 | 5 | 3 | 14 | 21 | 100 |
| Total | 46 | 7 | 275 | 40 | 41 | 6 | 100 | 14 | 24 | 3 | 66 | 10 | 54 | 8 | 86 | 12 | 692 | 100 |

Table S1. Index categories A05 Ordered mission and A06 Inconclusive problem distributed by ICPC-2 symptom categories
